# Serum from Adolescents with High Polyphenol Intake Exhibits Improved Lipid Profile and Prevents Lipid Accumulation in HepG2 Human Liver Cells

**DOI:** 10.1155/2023/1555942

**Published:** 2023-02-13

**Authors:** Giuseppina Augimeri, Ennio Avolio, Giovanna Caparello, Angelo Galluccio, Daniela De Rose, Adele Vivacqua, Catia Morelli, Ines Barone, Stefania Catalano, Sebastiano Andò, Cinzia Giordano, Diego Sisci, Stefania D'Angelo, Daniela Bonofiglio

**Affiliations:** ^1^Department of Pharmacy, Health and Nutritional Sciences, University of Calabria, Rende, 87036 Cosenza, Italy; ^2^Health Center Srl, 87100 Cosenza, Italy; ^3^Department of Clinical and Experimental Medicine, University Magna Graecia, 88100 Catanzaro, Italy; ^4^Centro Sanitario, University of Calabria, 87036 Arcavacata di Rende, Italy; ^5^Department of Movement Sciences and Wellbeing, University of Naples “Parthenope”, 80133 Naples, Italy

## Abstract

The traditional Mediterranean diet (MD) is characterized by a high phenolic-rich food intake, including in particular vegetables and fruits, but also legumes, whole grain cereals, nuts, and extra virgin olive oil. Evidence for beneficial effects of polyphenols in humans depends on the amount consumed and on their bioavailability. Here, we evaluated the association between the estimated polyphenol intake by fruits and vegetables food source and serum biochemical parameters in healthy adolescents, recruited into the DIMENU research project. Categorizing adolescents into three groups according to their estimated total polyphenol intake, we found that adolescents who declared high consumption of polyphenols had a higher adherence to the MD and had a better serum lipid profile than adolescents consuming low amounts of polyphenols. Moreover, using human HepG2 liver cells treated with oleic acid as an in vitro model for studying lipid accumulation, we showed that intracellular lipid accumulation is alleviated by serum from adolescents consuming a polyphenol-rich diet following MD recommendations. Our data underline the importance of promoting adherence to the typical MD foods as a superior strategy to prevent metabolic and chronic diseases and to ensure a better quality of life among adolescents.

## 1. Introduction

It is well recognized that nutrition plays an important role in health status; particularly, a high plant-based diet rich in fruits and vegetables may provide protective effects against many noncommunicable diseases (NCDs), such as cardiovascular diseases, type 2 diabetes, and some types of cancer [1–3]. In the past 10 years, a rise in interest in fruit and vegetable consumption has been motivated by their content of polyphenols [4]. Fruits like apples, grapes, pears, and berries typically have high amounts of polyphenols (200–300 mg per 100 g); also, vegetables, such as broccoli, carrots, and cabbages, contain a similar polyphenol content in fresh mass (100-300 mg per 100 g) [5, 6]. Apart from fruits and vegetables, other dietary sources of these phytochemicals include chocolate, tea, nuts, and olive oil as well as to a lesser extent dry legumes and whole grains [7]. Polyphenols are a very heterogeneous and widespread group of compounds, with more than 8000 different molecules characterized by the presence of one or more aromatic rings bearing hydroxyl groups [8]. Dietary polyphenols are divided into four classes: flavonoids, phenolic acids, stilbenes, and lignans, which can exist in a glycosidic form (glycosides of flavonoids, lignans, and stilbenes) or as esters (phenolic acids esterified to polyols such as quinic acid) [9]. Flavonoids can be classified into six subclasses based on the specific heterocyclic ring involved: flavonols, flavones, isoflavones, flavanones, anthocyanins, and flavanols (catechins and proanthocyanidins). In addition, two classes of phenolic acids can be distinguished into benzoic acid derivatives and cinnamic acid derivatives [7, 10, 11]. The most common phenolic acids are caffeic acid and ferulic acid, which are the major phenolic compounds in coffee and cereals, respectively. The best-studied stilbene is resveratrol in grapes, grape products, and red wine [10, 12].

These bioactive compounds are responsible for some sensory and health properties of foods, such as bitterness, astringency, and antioxidant ability. However, the considerable diversity of their structures makes polyphenols different from other antioxidants. Furthermore, the conjugation reactions with methyl, sulfate, or glucuronide groups as well as the nature and amounts of metabolites formed by the gut microflora may influence their absorption and their bioavailability [6, 13, 14]. In addition, the intake of these compounds and their food sources are highly variable and are linked with dietary patterns, sex, socioeconomic factors, and the native foods of each region.

Many studies, including epidemiologic cohort and case-control studies, as well as preclinical studies, have shown that the regular consumption of polyphenol-rich foods may reduce the incidence of obesity, metabolic syndrome, and liver disorders [15]. For example, polyphenols like catechins, resveratrol, and curcumin have been found to inhibit lipogenesis and enhance energy expenditure, leading to weight loss and antiobesogenic effects in different cell, animal, and human studies [16, 17]. Moreover, in human interventional trials, it has been widely demonstrated that polyphenols exert antioxidant and anti-inflammatory effects, preventing the progression of the metabolic syndrome [18].

Nonalcoholic fatty liver disease (NAFLD) is the most common chronic disease that may lead to severe pathologic conditions such as cirrhosis and hepatocellular carcinoma [19]. Adults as well as children with fatty liver display abnormal glucose and lipid metabolism [20]. The early events of NAFLD, studied in animals and in cell models of hepatic steatosis, including human hepatoblastoma HepG2 cell line [21], are triglyceride accumulation in the liver and insulin resistance, which is considerably affected by different causes such as hyperenergetic diets, sedentary lifestyle, and genetic factors. Fat accumulation in the liver is associated with lipotoxic hepatocellular injury due to elevated free fatty acids, free cholesterol, and other lipid metabolites. Thus, mitochondrial dysfunction with oxidative stress and endoplasmic reticulum stress-associated mechanisms are activated [22]. Currently, there is no agreement with respect to the pharmacological treatment of NAFLD, but lifestyle interventions based on exercise and a balanced diet for quality and quantity are considered the cornerstone of the NAFLD management [23].

The Mediterranean diet (MD), which is characterized by a high intake of vegetables, fruits, whole grain cereals, legumes, low-fat dairy, and extra virgin olive oil, has been suggested to decrease the risk of many NCDs, including liver disorders [24–27]. It is worth noting that the optimal adherence to the MD has been associated with the reduced risk of development and progression of NAFLD, due to the nutraceutical effects of bioactive compounds such as fibers, omega-3 fatty acids, vitamins, and polyphenols [28, 29]. Despite the promising evidence about the possible role of polyphenols in NCDs [30, 31], data regarding their consumption at the population level is not strong enough to recommend dietary intake levels [32]. Identifying an optimal polyphenol consumption is particularly relevant in the young population. Indeed, a healthy dietary pattern based on the consumption of polyphenol-based foods in adolescence might prevent the development of several NCD in adulthood. In order to estimate the dietary intake of polyphenols, several web databases have been developed, among which the web-based Phenol-Explorer database is the most comprehensive electronic tool on polyphenol contents. Indeed, Phenol-Explorer database, using information on food composition, metabolism, and pharmacokinetics of polyphenols, retrieves a reliable estimation on the polyphenol content from dietary sources [11, 33, 34].

The aim of this study was to estimate polyphenol intake from fruits and vegetables food source documented in a 24-hour dietary recall, by using the Phenol-Explorer database, in healthy adolescents. According to their estimated total polyphenol intake, adolescents were categorized into three different groups in order to evaluate the impact of polyphenol intake on serum metabolic profile. Furthermore, the potential properties of serum from adolescents were also investigated using an in vitro cell culture model of lipid accumulation.

## 2. Materials and Methods

### 2.1. Study Population

The population sample was recruited into the DIMENU research project (Mediterranean Diet and Swimming-Calabria FESR-FSE 2014-2020, prot. 52243/2017), in which adolescents were enrolled by the Castrolibero Institute of Education (Cosenza, Italy) and by sports associations of the Calabria region, Italy [35–38]. As part of the DIMENU project, in the current study, we investigated the total population of 56 subjects (31 girls and 25 boys) aged between 14 and 17 years. The exclusion criteria from the study included health problems, drug use, supplement intake, any type of restrictive diet (i.e., low calorie, low carb, and low fat content) and cognitive, physical, or motor limitation. Ethical approval was obtained by the Ethics Committee of the University of Calabria, Italy (# 5727/2018) to conduct this study.

### 2.2. Anthropometric Parameters and Physical Activity Intensity Levels

In all participants, anthropometric data were collected using a validated protocol [39], in particular by measuring height and bodyweight; the body mass index (BMI) was calculated as previously reported [38]. Physical activity levels, based on the WHO recommendations [40], were classified according to the metabolic equivalents (METs), particularly physical inactivity (<3 metabolic equivalents (METs), moderate PA (3 to 6 METs), and vigorous-intensity PA (>6 METs), using a questionnaire that we have described elsewhere [38].

### 2.3. Adherence to the Mediterranean Diet by KIDMED Test and Dietary Assessment by 24 h Recall

The KIDMED test was used to assess the adherence to the MD, providing a score ranging from <0 to ≤12 according to the 16-point questions (12 positive and 4 negative items) [41]. Nutritionists collected daily meals from each subject through an in-depth interview of the 24-hour recall which investigated detailed data about food preparation methods, ingredients used, and amounts of each food consumed in reference to a common size container (e.g., bowls, cups, and glasses). The dietary survey was carried out the day before the interview and planned excluding the investigation of the food intake referring to the weekend. A photographic manual of portion sizes was used to visually estimate food amounts. Nutrient intakes were calculated by multiplying the portion weight by its nutrient content. Specific software MetaDieta software version 4.2.1. (Meteda s.r.l., Roma, Italy), which includes the Italian database, was used to analyze the energy and nutrient content of food intake.

### 2.4. Biochemical and Hormonal Measurements

After a 12-hour overnight fasting, venous blood samples were collected and centrifuged in order to obtain serum. The biochemical parameters were analyzed by a Konelab 20i chemistry analyzer (Thermo Electron Corporation, Vantaa, Finland) according to standard procedures. Serum C-reactive protein (CRP) levels were determined by immunonephelometry (GOLDSITE Diagnostics, Inc., Shenzhen, China). Serum insulin levels were detected with an enzyme-linked immunosorbent assay (ELISA) kit (NovaTec Immundiagnostica GmbH, Dietzenbach, Germany) following the manufacturer's instructions. The lowest detectable concentration of insulin was 0.25 *μ*IU/mL at a 95% confidence limit; the intra-assay variability was within ≤5%. Homeostasis model assessment for estimating insulin resistance (HOMA-IR) which was calculated as the product of fasting glucose concentration (mg/dL) and fasting insulin concentration was divided by 405. Erythrocyte sedimentation rate (ESR) was measured by the Wintrobe method.

### 2.5. Phenol-Explorer Database

Phenol-Explorer is an updated comprehensive database for natural polyphenols including food synthesis, processing, and humans' polyphenol metabolites (http://phenol-explorer.eu-version3.6). The last version of Phenol-Explorer database provides data on 502 polyphenol compounds in 452 plant-based foods collected from 638 scientific peer-reviewed articles and divided in the main polyphenol classes [42–44]. The Phenol-Explorer 3.6 database reports data that also consider the effects of cooking and food processing on polyphenol content. The data from total polyphenols in the database was expressed as total phenolics, which was determined by using the Folin-Ciocalteu assay; the value of the individual polyphenols was evaluated by chromatography [44, 45]. The only exception to the use of this method was for determination of total anthocyanins, using the pH differential method [46]. Caution should be taken when comparing retention factors obtained by different methods, as polyphenols degraded during food cooking or processing may no longer be detectable by HPLC, but still keep an absorbance or ability to reduce the Folin reagent [44]. Total polyphenol content was calculated as the sum of the contents of individual compounds expressed in mg/100 g food fresh weight [45].

### 2.6. Estimation of Dietary Polyphenol Intake

The calculation of polyphenol intake was derived from matching the dietary assessment by 24 h recall and food (fruits and vegetables) data in the Phenol-Explorer database. The fruit and vegetable intakes were calculated (in g) by following the portions sizes reported by each subject. An advanced search was carried out in the Phenol-Explorer database to retrieve mean content values for total polyphenols contained in the foods obtained. Total polyphenol intake was calculated as the sum of all individual polyphenol intakes from all food sources reported [13, 33, 34, 45].

### 2.7. Antioxidant Ability Assessed in Serum

#### 2.7.1. FRAP Assay

The ferric reducing antioxidant power (FRAP) method measures the change in absorbance that occurs when the TPTZ (2,4,6-tris-pyridyl-S-triazine) (–Fe(III)) complex is reduced to the TPTZ-Fe2+ form in the presence of antioxidant compounds [47]. Briefly, the FRAP reagent was prepared by mixing 10 mM tris-pyridyl-S-triazine (TPTZ) solution in 40 mM HCl plus 20 mM FeCl3 and 0.25 M sodium acetate buffer (pH 3.6) in a volume ratio of 1 : 1 : 10. The acetate buffer used in the FRAP assay was prepared by dissolving 1.90 g/L of sodium acetate in water and adjusted to pH 3.6 with 16.0 mL/L glacial acetic acid. An aliquot (6 *μ*L) of serum from adolescents stratified in three groups according to their polyphenol intake was mixed with 140 *μ*L of FRAP reagent and 0.06 *μ*L of H_2_O. The absorbance of the reaction mixture was measured at 593 nm in Multiskan SkyHigh (Thermo Fisher Scientific, Waltham, MA, USA). A solution of FeSO_4_ (1 mM) was used to obtain the calibration curve. 2,4,6-Tris-pyridyl-S-triazine (TPTZ), FeCl_3_, FeSO_4_, glacial acetic acid, and oleic acid were purchased from Sigma-Aldrich (Milan, Italy).

#### 2.7.2. DPPH Assay

The 2,2-diphenyl-1-picrylhydrazyl (DPPH, Sigma-Aldrich) assay is based on the reduction of the purple DPPH• to 1,1-diphenyl-2-picryl hydrazine by antioxidant compounds [48]. Serum aliquots (100 *μ*L) were mixed with 100 *μ*L of methanol, incubated for 2 min, and then centrifuged (10 min, 48°C, 9500 = g). Supernatant samples were immediately tested for the DPPH radical scavenging activity. Briefly, 10 *μ*L of serum sample was mixed with 100 *μ*L of a 0.1 mM DPPH solution in absolute methanol and incubated in darkness at room temperature for 30 min. The absorbance was read at 520 nm in Multiskan SkyHigh (Thermo Fisher Scientific). The sample absorbance was compared with the absorbance of a control containing only methanol and DPPH solution. The percentage of DPPH inhibition was calculated using the following equation:

(1) $$\begin{array}{c} \%\text{IDPPH}=\left[1-\left(\frac{\text{As}}{\text{A}0}\right)\right]*100, \end{array}$$

where As is the absorbance of sample and A0 is the DPPH solution absorbance.

### 2.8. Cell Culture and Experimental Treatments

Human hepatocellular carcinoma HepG2 cells were obtained from American Type Culture Collection (ATCC, USA) and authenticated and stored according to the supplier's instruction. HepG2 cells were cultured in Eagle's Minimum Essential Medium (EMEM) (ATCC) and supplemented with 10% fetal bovine serum (FBS, Life Technologies) and 1% penicillin-streptomycin (Sigma-Aldrich) at 37°C in a humidified 5% CO2 atmosphere. For staining of lipid droplets with Oil Red O, 140,000 HepG2 cells were seeded in 24-multi-well dishes for 24 hours. Subsequently, the cells were pretreated for 1 hour in serum-free medium with 10% of pooled serum of all adolescents as control and pooled serum from adolescents with low, medium, and high polyphenol intake before exposure of 0.1 mM oleic acid (OA) for 24 hours.

### 2.9. MTT Assay

HepG2 cells were seeded in a 24-well plate and exposed to different concentration (0.1-1 mM) of OA for 24 hours. Cell viability was determined with the 3-(4,5-dimethylthiazol-2-yl)-2,5-diphenyltetrazolium (MTT) assay, as previously described [49]. The results were expressed as a percentage of viable cells in comparison to the control (taken as 100%).

### 2.10. Staining of Lipid Droplets with Oil Red O

After incubation with oleic acid, HepG2 cells were washed with PBS and fixed with 4% paraformaldehyde for 30 minutes at room temperature. Then, the cells were stained with Oil Red O (Bio Optica, Milan, Italy) as described in the manufacturer's protocol and observed under a light microscope (Olympus). To quantify Oil Red O content, the cells were treated with 100% isopropanol and lipid accumulation was evaluated at 510 nm in Multiskan SkyHigh (Thermo Fisher Scientific).

### 2.11. Statistical Analysis

Data were reported as the mean and standard deviation (SD) or standard error of mean (SEM) as indicated.  Table 1 shows the average values and SD of total cholesterol, low-density lipoprotein (LDL), and triglycerides (TG) along with the effect size, the corresponding 95% confidence intervals, and the *p* values, evaluated by equivalence test, between low and high polyphenol intake groups. Statistical differences between groups were evaluated by using parametric tests (one-way ANOVA and Student's *t*-test). The correlation between variables was evaluated by Spearman's correlation test. Statistical significance was set at *p* < 0.05.

## 3. Results

### 3.1. Characteristics of Participants and Total Polyphenol Intake

The general characteristics of the study population are presented in Table 2. A total of 56 adolescents with a mean age of 15.87 (±1.04) years old were included in the final analysis, specifically 31 girls and 25 boys. Normal mean values of BMI were found in our population (22.96 ± 3.33). We also reported the data on the three intensity levels of the physical activity of participants, according to the guidelines established by the WHO [39]. In addition, the KIDMED test revealed a score < 4.52 (1^st^ tertile), within 4.52-7.38 (2^nd^ tertile) and >9.52 (3^rd^ tertile) and a mean score of 7.14 (±2.23), indicating an average adherence to the Mediterranean diet (MD) in our adolescents. The mean daily intake of total polyphenols was estimated at 434.46 (±514.27) mg/day, consisting of approximately 49% of flavonoids, 39% of phenolic acids, and 12% of other polyphenols. No differences were observed in the general characteristics according to sex (data not shown).

### 3.2. Total Polyphenol Intake Associates with Mediterranean Diet Adherence in Adolescents

Previous studies showed that the polyphenol intake is positively associated with the adherence to the Mediterranean diet in an adult Italian population [50], but there is no adequate data about this correlation in healthy adolescents. Testing the association between the total estimated polyphenol intake and the adherence to the MD in all adolescents, we found a significant linear increasing association (*r* = 0.411 and *p* = 0.001). We did not find any difference in the polyphenol intake according to sex, physical activity, or BMI (data not shown).

### 3.3. Mediterranean Diet Adherence and Dietary and Food Intakes of Adolescents Stratified according to Their Estimated Total Polyphenol Intake

Based on daily total polyphenol intake, we classified the study participants into the low, moderate, and high polyphenol intake groups (Table 3). We found that adolescents allocated in the highest tertile of total polyphenol consumption had significantly higher adherence to the MD than participants from the lowest tertile (8.21 ± 1.84 vs. 6.15 ± 2.22, *p* = 0.01).

Using a 24-hour dietary recall method, we evaluated the mean daily intakes of energy, macronutrients, and micronutrients in our adolescents grouped according to the estimated total polyphenol intake. As shown in Table 4, no statistically significant differences were found among the three groups in absolute intakes of energy and most macronutrients. However, we observed that adolescents with high polyphenols' consumption showed greater intakes of omega-3 fatty acids and soluble and insoluble dietary fibers than those with low polyphenols' consumption group. The same differences were revealed after energy adjustment (data not shown). As regarding the intakes of micronutrients, the mean levels of vitamin B5, B6, B8, B12, K, phosphorus, magnesium, selenium, potassium, and copper were increased in high compared to low consumers. Similarly, vitamins and minerals, except phosphorus and copper, displayed the significant differences after energy adjustment (data not shown). In addition, intake of water was greater in high than low and moderate polyphenol intake groups. Interestingly, the dietary total antioxidant capacity evaluated by the Oxygen Radical Absorbance Capacity (ORAC) showed increased total ORAC in adolescents with high compared with low intake group.

### 3.4. Antioxidant Activity of Serum Samples from Adolescents Classified according to their Estimated Total Polyphenol Consumption

**Figure 1:**
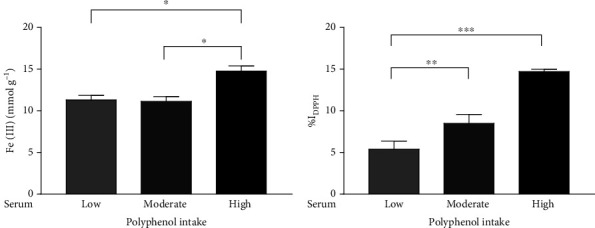
Antioxidant capacity assessed by ferric reducing ability (FRAP) assay (a) and percentage of inhibition against DPPH (b) in the pool of serum from adolescents with low, moderate, and high polyphenol intake (low, moderate, and high). The bar graphs showed the mean ± SEM of 3 independent experiments. ^∗^*p* < 0.05, ^∗∗^*p* < 0.005, and ^∗∗∗^*p* < 0.001.

Polyphenols have been largely studied for their effects on human health due to their potential antioxidant properties, which decrease the risk of several diseases, including metabolic disorders and cardiovascular and liver diseases [29, 51, 52]. Based on our results showing that the dietary total ORAC increased in high polyphenol consumers, we evaluated the potential antioxidant properties of serum samples from the adolescents enrolled in this study. We found that serum from high consumers displayed a significantly enhanced ferric reducing antioxidant activity than that from adolescents consuming moderate and low amounts of polyphenols (Figure 1(a)). Consistent with these results, we also observed that pooled serum from adolescents consuming high amounts of polyphenols exhibited a higher percentage of inhibition against DPPH compared to the moderate and low polyphenol consumers (Figure 1(b)).

### 3.5. Biochemical, Metabolic, and Inflammatory Serum Profile from Adolescents Classified according to Their Estimated Total Polyphenol Intake

Serum levels of general, metabolic, and inflammatory biomarkers in subjects classified according to the tertiles of total polyphenols' consumption were evaluated, since no significant association between total polyphenol intake and serum biomarkers data was observed in all adolescents (Table 5). Although normal values of circulating biomarkers were found in our population sample, we observed significantly reduced serum levels of triglycerides (TG), total cholesterol, and low-density lipoprotein (LDL) along with ESR values in adolescents who declared high consumption of polyphenols.

### 3.6. Effects of Serum from Adolescents with Different Estimated Polyphenol Intake against Oleic Acid-Induced Lipid Accumulation in Human HepG2 Liver Cells

**Figure 2:**
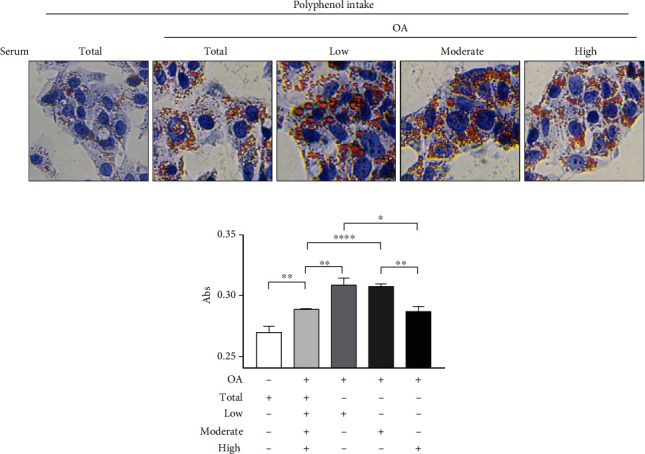
Lipid accumulation in human HepG2 liver cells treated with oleic acid in the presence of serum from adolescents. (a) HepG2 cells were pretreated with the pool of serum from all adolescents (total) or with the pool of serum from low, moderate, and high polyphenols' consumers (10% *v*/*v*), followed by the treatment with oleic acid (OA) 0.1 mM for 24 hours. Lipid accumulation was observed by Oil Red O staining. (b) HepG2 cells were treated with isopropanol, and the quantification of intracellular lipid accumulation was measured at 510 nm. The bar graphs showed the mean ± SEM of 3 independent experiments, each performed in triplicate. ^∗^*p* < 0.05, ^∗∗^*p* < 0.005, and ^∗∗∗∗^*p* < 0.0001.

In order to investigate the properties of the serum from adolescents with different polyphenols' consumption in the prevention of the liver damage, we used human hepatocyte-derived cell line HepG2 exposed to oleic acid (OA), as the main alternative in vitro model for studying lipid accumulation. First, in order to evaluate the effects of OA on HepG2 cell viability, MTT assay was performed. We observed that OA at 0.1 mM did not display any cell toxicity (data not shown). Thus, we pretreated the cells with 10% of pooled serum from all adolescents as control and pooled serum from adolescents with low, moderate, and high polyphenol intake, and we induced lipid accumulation with OA 0.1 mM for 24 hours (Figure 2(a)). Interestingly, serum from high polyphenol consumers reduced lipid accumulation compared to adolescents with low or moderate polyphenol intakes, as revealed by the quantification of intracellular lipid content, addressing the role of polyphenol-rich diet in the prevention of lipid accumulation (Figure 2(b)).

## 4. Discussion

**Figure 3:**
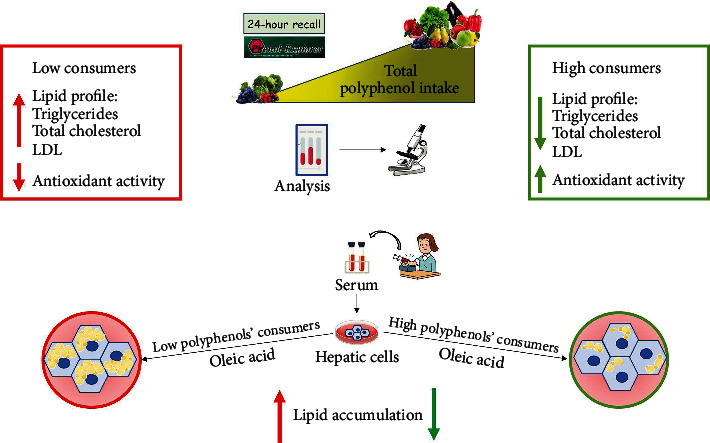
Graphical representation of the main results of our study.

In this study, we evidenced that serum from adolescents who declared a high intake of polyphenols displays antioxidant properties and protects against in vitro hepatic lipid accumulation, supporting the idea that a polyphenol-rich diet may have an important role in the prevention of metabolic and chronic diseases. Firstly, fruits and vegetables reported in 24-hour dietary recalls and the Phenol-Explorer database were matched, and the estimated total polyphenol intake in our population was calculated. Among adolescents enrolled in our study, the estimated intake of polyphenols was a mean of 434.46 mg/day, consisting of 49% of flavonoids, 39% of phenolic acids, and 13% of other polyphenols. These results are in agreement with the data described in the HELENA study investigating the dietary intake of polyphenols in European adolescents [53]. Analyzing the relationship between the estimated total polyphenol intake and the general characteristics of our population, we did not find any significant correlation between the consumption of polyphenols and sex, physical activity, or BMI. Interestingly, we evidenced a significant linear positive association between total polyphenol intake and the adherence to the MD. In particular, stratifying the adolescents into three groups based on their polyphenol intake (low, moderate, and high), KIDMED score revealed an optimal adherence to the MD (8.21 ± 1.84) in high polyphenols' consumers, suggesting that the health benefits of the MD could be related to the high intake of fruits and vegetables. Other authors have reported a linear correlation between MD and the intake of polyphenols [42, 50]. Nowadays, the MD is considered one of the healthiest eating patterns, due to high consumption of fruits and vegetables, which represent the main food sources of polyphenols, along with a high intake of legumes, nuts and whole grains, low-fat dairy, a moderate intake of fish, and reduced consumption of meat [54], thus providing a balanced intake of macro- and micronutrients. Analyzing the dietary intake assessment by 24-hour recall interview, the primary energy sources in foods did not reflect the optimal proportions of daily intakes even though total energy intake is in the range of the suggested dietary intake allowances in our population according to the total polyphenol intake, suggesting a same quantitative dietary pattern among adolescents. However, in spite of the unchanged total energy intakes, we found qualitative differences in several nutrients among the three groups, which levels are in the average of the recommended allowances. In particular, the intakes of soluble and insoluble fibers and omega-3 fatty acids along with several vitamins and minerals significantly increased in high compared to low polyphenols' consumers, showing a good eating behavior and a better compliance with the MD pyramid recommendations. It is worth noting that in our adolescents, the primary energy sources in foods did not reflect the optimal proportions of daily intakes. The MD emphasizes eating foods like vegetables and fruits because of their elevated micronutrient content, in terms of vitamins, minerals, and phytochemicals, which are able to elicit different beneficial effects, including antioxidant properties. In our population, total antioxidant capacity from the diet evaluated by ORAC values resulted increased in adolescents who consume high amounts of polyphenols. These data correlated with the antioxidant power of the serum samples of our adolescents, measured by conventional total antioxidant assay methods. Those methods, based on the radical scavenging activity and redox potential of antioxidants, displayed in high polyphenol intake group an overall serum antioxidant capacity and predicted the body's antioxidant status. Another favorable effect of polyphenols is related to their ability in ameliorating lipid/lipoprotein metabolism and alleviating hyperlipidemia [55]. However, although the common consumption of polyphenol-rich diet would maintain or improve the lipid profile of healthy participants [56–59], further studies are needed to clarify the preventive actions of these phytochemicals in healthy individuals. In addition, a crucial aspect that has to be investigated is to distinguish the specific effects of polyphenols versus those of many prohealth components (e.g., vitamins, fibers, and minerals) present in fruits and vegetables. Although we failed to observe a significant association between total polyphenol intake and serum biomarkers in all adolescents, maybe due to the small sample investigated, our data revealed a better lipid profile, in terms of reduced serum concentration of TG, total cholesterol, and LDL in healthy adolescents with high polyphenol intake. An imbalance between lipid acquisition and lipid disposal leads to a hepatic fat accumulation, which is of pivotal importance in the development of NAFLD, and it is also a risk factor for many other chronic metabolic diseases. Using an in vitro model of lipid accumulation represented by the OA-induced human HepG2 liver cells, we showed that intracellular lipid accumulation is alleviated by serum from adolescents consuming a polyphenol-rich diet and following MD recommendations. Although we cannot unravel whether polyphenol intake caused the lipid biomarkers or whether the lipid biomarkers led to changes in the diet in our study, we may speculate the benefits of healthy eating pattern in the complex pathology of NAFLD. Collectively, our findings are graphically reported in Figure 3.

This study includes some limitations, such as the relatively small sample size, particularly when the total sample was divided into the three groups, as well as the lack of direct methods to measure polyphenol serum levels. However, data from clinical setting together with experimental cell model strengthen the importance of improving healthy dietary habits in adolescents in an attempt to achieve clinical health outcomes.

## 5. Conclusions

Although further studies and deeper knowledge about polyphenol intakes and several prohealth components from the MD pattern might be helpful for planning targeted prevention strategies at an early age, we support and encourage the Mediterranean eating style for the prevalent fruit and vegetable consumption which should represent a protective choice against the development of NCDs, including NAFLD.

## Figures and Tables

**Table 1:** The differences of total cholesterol, low-density lipoprotein cholesterol, and triglycerides values between low and high polyphenol intake (PI) groups.

Biomarkers	Average values and SD (mg/dL)	Effect size (mg/dL)	95% confidence interval (mg/dL)	*p* values
Total cholesterol	Low PI: 163.2 ± 37.1 High PI: 140.3 ± 20.6	22.9	3.0-42.9	0.025
LDL	Low PI: 93.3 ± 27.5 High PI: 74.9 ± 17.1	18.4	3.1-33.5	0.020
TG	Low PI: 112.1 ± 109.7 High PI: 55.6 ± 15.3	56.5	3.2-109.7	0.039

LDL: low-density lipoprotein; TG: triglycerides; PI: polyphenol intake; SD: standard deviation.

**Table 2:** General characteristics of all participants.

Total population (number of subjects)	56
Girls/boys (numbers)	31/25
Age (years)	15.87 ± 1.04
BMI (kg/m^2^)	22.96 ± 3.33
BMI (kg/m^2^) percentiles	73.51 ± 20.81
Physical activity intensity levels (number of subjects)	
Sedentary	14
Moderate	21
Vigorous	21
KIDMED score	7.14 ± 2.23
Total polyphenol intake (mean mg/day)	434.46 ± 514.27
Flavonoids (%)	49
Phenolic acids (%)	39
Other polyphenols (%)	12

BMI: body mass index. Data are expressed as mean ± SD.

**Table 3:** KIDMED score in adolescents grouped according to their total estimated total polyphenol consumption.

	Total polyphenol intake (mg/day)			
	Low (*n* = 19)	Moderate (*n* = 18)	High (*n* = 19)	*p* value
	<210	210-460	>460	^∗^0.32 **¥ 0.0001 § 0.0002**
KIDMED score	6.15 ± 2.22	7.05 ± 2.23	8.21 ± 1.84	^∗^0.40 **¥ 0.01** § 0.23

^∗^: low vs. moderate; ¥: low vs. high; §: moderate vs. high. Data are expressed as mean ± SD. Statistical differences were evaluated by a one-way ANOVA test. In bold are reported statistically significant values.

**Table 4:** Energy and nutrient intakes from 24-hour recall in adolescents stratified with respect to the total polyphenol intake.

	Total polyphenol intake			
	Low	Moderate	High	*p* value
Primary energy sources				
Total energy (kcal)	1656.10 ± 426.74	1713.22 ± 517.21	1567.47 ± 408.86	^∗^ 0.92 ¥ 0.82 § 0.59
Total energy (kJ)	6929.144 ± 1785.48	7168.12 ± 2164.01	6558.31 ± 1710.67	^∗^ 0.92 ¥ 0.82 § 0.59
Total fat (g)	79.80 ± 20.78	77.49 ± 26.05	71.33 ± 33.74	^∗^ 0.96 ¥ 0.61 § 0.77
Total carbohydrate (g)	162.46 ± 81.63	180.12 ± 76.34	146.68 ± 49.93	^∗^ 0.73 ¥ 0.77 § 0.33
Total protein (g)	65.01 ± 20.66	71.36 ± 28.08	77.19 ± 21.75	^∗^ 1 ¥ 0.44 § 0.45
Animal protein (g)	37.63 ± 23.88	39.78 ± 23.52	49.60 ± 30.68	^∗^ 0.97 ¥ 0.35 § 0.50
Vegetable protein (g)	22.03 ± 10.04	23.66 ± 11.35	20.41 ± 8.10	^∗^ 0.87 ¥ 0.87 § 0.58
Fats				
SFA (g)	22.60 ± 11.33	20.03 ± 8.71	17.46 ± 10.90	^∗^ 0.73 ¥ 0.29 § 0.73
MUFA (g)	39.32 ± 14.09	40.48 ± 14.10	34.99 ± 20.80	^∗^ 0.95 ¥ 0.70 § 0.60
PUFA (g)	6.90 ± 2.56	8.20 ± 2.91	9.42 ± 6.27	^∗^ 0.62 ¥ 0.17 § 0.66
Vegetable fats (g)	46.95 ± 9.81	47.37 ± 18.85	41.24 ± 16.76	^∗^ 0.99 ¥ 0.51 § 0.47
Animal fats (g)	24.84 ± 22.19	21.91 ± 14.76	29.59 ± 30.18	^∗^ 0.92 ¥ 0.81 § 0.58
Omega-3 fatty acids (g)	0.84 ± 0.30	1.05 ± 0.55	1.57 ± 1.21	^∗^ 0.75 ¥ **0.02** § 0.12
Omega-6 fatty acids (g)	6.16 ± 2.10	6.45 ± 2.46	7.39 ± 5.42	^∗^ 0.97 ¥ 0.57 § 0.72
EPA (g)	0.05 ± 0.11	0.10 ± 0.20	0.16 ± 0.26	^∗^ 0.78 ¥ 0.23 § 0.58
DHA (g)	0.08 ± 0.18	0.13 ± 0.28	0.44 ± 0.94	^∗^ 0.96 ¥ 0.15 § 0.26
PUFA : SFA ratio	0.23 ± 0.21	0.22 ± 0.10	0.34 ± 0.24	^∗^ 0.99 ¥ 0.20 § 0.18
Cholesterol (mg)	182.10 ± 122.05	182.33 ± 119.99	249.63 ± 199.50	^∗^ 1 ¥ 0.36 § 0.38
Carbohydrates				
Starch (g)	96.80 ± 53.65	110.69 ± 62.37	79.80 ± 42.41	^∗^ 0.71 ¥ 0.59 § 0.19
Soluble sugars (g)	48.43 ± 36.01	51.71 ± 23.75	60.48 ± 20.79	^∗^ 0.93 ¥ 0.38 § 0.60
Glycemic index	60.50 ± 19.55	63.51 ± 18.10	53.70 ± 7.96	^∗^ 0.84 ¥ 0.40 § 0.16
Glycemic load	88.50 ± 66.71	93.57 ± 60.09	65.17 ± 27.00	^∗^ 0.96 ¥ 0.38 § 0.25
Fibers				
Total dietary fiber	12.95 ± 4.46	15.19 ± 6.51	15.21 ± 4.93	^∗^ 0.42 ¥ 0.40 § 1
Soluble dietary fiber	1.70 ± 1.14	2.47 ± 1.38	2.91 ± 1.50	^∗^ 0.20 ¥ **0.02** § 0.59
Insoluble dietary fiber	4.54 ± 2.46	7.75 ± 3.80	8.18 ± 4.15	^∗^ **0.02** ¥ **0.007** § 0.93
Vitamins				
Vitamin A eq. retinol (*μ*g)	974.91 ± 717.13	1044.16 ± 624.45	1274.02 ± 686.88	^∗^ 0.95 ¥ 0.38 § 0.56
Vitamin B1 (mg)	1.15 ± 1.67	0.76 ± 0.29	0.87 ± 0.31	^∗^ 0.47 ¥ 0.66 § 0.95
Vitamin B2 (mg)	1.03 ± 0.47	1.17 ± 0.43	1.25 ± 0.55	^∗^ 0.67 ¥ 0.38 § 0.88
Vitamin B3 (mg)	15.79 ± 7.92	14.81 ± 7.73	17.65 ± 8.00	^∗^ 0.92 ¥ 0.75 § 0.52
Vitamin B5 (mg)	1.88 ± 1.49	2.43 ± 1.87	3.26 ± 1.70	^∗^ 0.59 ¥ **0.04** § 0.30
Vitamin B6 (mg)	1.45 ± 0.62	1.61 ± 0.60	2.06 ± 0.55	^∗^ 0.69 ¥ **0.008** § 0.06
Vitamin B8 (*μ*g)	8.13 ± 5.93	12.34 ± 9.60	18.27 ± 13.97	^∗^ 0.43 ¥ **0.01** § 0.20
Folic acid (*μ*g)	228.15 ± 100.30	250.29 ± 114.85	222.56 ± 91.12	^∗^ 0.79 ¥ 0.98 § 0.69
Vitamin B12 (*μ*g)	1.98 ± 1.69	3.48 ± 3.13	4.41 ± 2.30	^∗^ 0.16 ¥ **0.01** § 0.48
Vitamin C (mg)	124.66 ± 176.75	98.81 ± 48.80	132.91 ± 83.16	^∗^ 0.78 ¥ 0.97 § 0.64
Vitamin K (*μ*g)	1.33 ± 2.49	2.69 ± 4.26	6.11 ± 8.82	^∗^ 0.76 ¥ **0.04** § 0.19
Vitamin D (*μ*g)	1.42 ± 1.80	1.66 ± 1.72	3.24 ± 4.91	^∗^ 0.97 ¥ 0.21 § 0.30
Vitamin E (mg)	12.67 ± 1.95	13.29 ± 4.34	13.16 ± 3.67	^∗^ 0.87 ¥ 0.87 § 0.58
Total ORAC (*μ*mol TE)	3844.89 ± 2347.86	4908.39 ± 2772.36	7862.79 ± 7217.15	^∗^ 0.77 ¥ 0.03 § 0.14
Minerals				
Calcium (mg)	412.11 ± 196.85	560.11 ± 257.10	630.17 ± 296.5	^∗^ 0.87 ¥ 0.87 § 0.58
Phosphorus (mg)	738.68 ± 341.02	948.54 ± 397.10	1014.03 ± 296.91	^∗^ 0.17 ¥ **0.04** § 0.83
Iodium (*μ*g)	69.80 ± 142.01	69.22 ± 81.78	95.53 ± 76.28	^∗^ 0.99 ¥ 0.73 § 0.73
Sodium (mg)	1356.37 ± 1161.84	1000.35 ± 859.83	1180.03 ± 1087.92	^∗^ 0.56 ¥ 0.82 § 0.86
Iron (mg)	7.32 ± 2.10	7.92 ± 2.85	8.95 ± 3.36	^∗^ 0.80 ¥ 0.19 § 0.51
Magnesium (mg)	134.95 ± 81.02	226.42 ± 155.94	223.36 ± 71.31	^∗^ **0.04** ¥ **0.04** § 0.99
Selenium (*μ*g)	16.09 ± 12.34	37.98 ± 44.53	67.91 ± 60.71	^∗^ 0.31 ¥ **0.002** § 0.11
Potassium (mg)	2086.14 ± 717.62	2609.25 ± 939.23	2743.79 ± 460.26	^∗^ 0.08 ¥ **0.02** § 0.84
Zinc (mg)	8.39 ± 5.22	8.13 ± 3.03	9.81 ± 4.23	^∗^ 0.98 ¥ 0.56 § 0.46
Copper (mg)	0.61 ± 0.39	0.86 ± 0.57	1.19 ± 0.90	^∗^ 0.49 ¥ **0.02** § 0.27
Water (g)	722.58 ± 348.85	783.53 ± 230.44	1096.57 ± 522.33	^∗^ 0.88 ¥ 0.01 § 0.05

SFA: saturated fatty acid; MUFA: monounsaturated fatty acid; PUFA: polyunsaturated fatty acid; EPA: eicosapentaenoic acid; DHA: docosahexaenoic acid; ORAC: Oxygen Radical Absorbance Capacity; eq.: equivalent. ^∗^: low vs. moderate; ¥: low vs high; §: moderate vs. high. Statistical differences were evaluated by a one-way ANOVA test. In bold are reported statistically significant values.

**Table 5:** Serum biomarkers in adolescents stratified with respect to the estimated total polyphenol intake.

	Total polyphenol intake			
	Low	Moderate	High	*p* value
Serum biomarkers				
Glucose (mg/dL)	80.79 ± 8.30	78.22 ± 7.53	78.21 ± 5.53	^∗^ 0.53 ¥ 0.51 § 1
Insulin (mU/L)	10.38 ± 6.68	10.50 ± 6.85	8.73 ± 6.26	^∗^ 1 ¥ 0.69 § 0.67
HOMA-IR	2.09 ± 1.16	2.04 ± 1.36	1.69 ± 1.23	^∗^ 0.99 ¥ 0.60 § 0.68
TG (mg/dL)	112.05 ± 109.72	67.33 ± 31.91	55.63 ± 15.28	^∗^ 0.11 ¥ **0.03** § 0.86
Total cholesterol (mg/dL)	163.21 ± 37.06	154.11 ± 27.44	140.26 ± 20.56	^∗^ 0.61 ¥ **0.05** § 0.33
LDL (mg/dL)	93.26 ± 27.54	91.89 ± 22.76	74.95 ± 17.06	^∗^ 0.98 ¥ **0.04** § 0.07
HDL (mg/dL)	47.47 ± 10.13	48.89 ± 12.90	54.26 ± 10.66	^∗^ 0.92 ¥ 0.16 § 0.32
Creatinine (mg/dL)	0.92 ± 0.11	0.87 ± 0.12	0.87 ± 0.12	^∗^ 0.45 ¥ 0.42 § 1
Urea nitrogen (mg/dL)	28.74 ± 5.69	28.28 ± 6.05	31.53 ± 6.68	^∗^ 0.97 ¥ 0.35 § 0.25
Uric acid (mg/dL)	4.90 ± 1.16	4.88 ± 1.80	5.08 ± 1.56	^∗^ 1 ¥ 0.93 § 0.91
Total bilirubin (mg/dL)	0.99 ± 0.62	0.88 ± 0.37	1.21 ± 0.75	^∗^ 0.85 ¥ 0.49 § 0.22
Direct bilirubin (mg/dL)	0.27 ± 0.09	0.24 ± 0.07	0.29 ± 0.11	^∗^ 0.64 ¥ 0.75 § 0.25
ESR (mm/h)	18.74 ± 7.06	24.72 ± 16.22	13.58 ± 7.95	^∗^ 0.24 ¥ 0.33 **§ 0.01**
CRP (mg/L)	1.27 ± 0.81	1.23 ± 0.64	1.12 ± 0.53	^∗^ 0.98 ¥ 0.77 § 0.87

HOMA-IR: Homeostasis model assessment for estimating insulin resistance; TG: triglyceride; LDL: low-density lipoprotein; HDL: high-density lipoprotein; ESR: erythrocyte sedimentation rate; CRP: serum C-reactive protein. ^∗^: low vs. moderate; ¥: low vs. high; §: moderate vs. high. Statistical differences were evaluated by a one-way ANOVA test. In bold are reported statistically significant values.

## Data Availability

The data used to support the findings of this study are available from the corresponding author upon request.

## References

[1] Grosso G., Bella F., Godos J. (2017). Possible role of diet in cancer: systematic review and multiple meta-analyses of dietary patterns, lifestyle factors, and cancer risk. *Nutrition Reviews*.

[2] Meccariello R., D'Angelo S. (2021). Impact of polyphenolic-food on longevity: an elixir of life. An overview. *Antioxidants*.

[3] Schwedhelm C., Boeing H., Hoffmann G., Aleksandrova K., Schwingshackl L. (2016). Effect of diet on mortality and cancer recurrence among cancer survivors: a systematic review and meta-analysis of cohort studies. *Nutrition Reviews*.

[4] Fraga C. G., Croft K. D., Kennedy D. O., Tomas-Barberan F. A. (2019). The effects of polyphenols and other bioactives on human health. *Food & Function*.

[5] Cieślik E., Gręda A., Adamus W. (2006). Contents of polyphenols in fruit and vegetables. *Food Chemistry*.

[6] Scalbert A., Manach C., Morand C., Remesy C., Jimenez L. (2005). Dietary polyphenols and the prevention of diseases. *Critical Reviews in Food Science and Nutrition*.

[7] Pandey K. B., Rizvi S. I. (2009). Plant polyphenols as dietary antioxidants in human health and disease. *Oxidative Medicine and Cellular Longevity*.

[8] Del Rio D., Rodriguez-Mateos A., Spencer J. P., Tognolini M., Borges G., Crozier A. (2013). Dietary (poly)phenolics in human health: structures, bioavailability, and evidence of protective effects against chronic diseases. *Antioxidants & Redox Signaling*.

[9] Tsao R. (2010). Chemistry and biochemistry of dietary polyphenols. *Nutrients*.

[10] Manach C., Scalbert A., Morand C., Remesy C., Jimenez L. (2004). Polyphenols: food sources and bioavailability. *The American Journal of Clinical Nutrition*.

[11] Perez-Jimenez J., Neveu V., Vos F., Scalbert A. (2010). Identification of the 100 richest dietary sources of polyphenols: an application of the phenol-explorer database. *European Journal of Clinical Nutrition*.

[12] Guasch-Ferre M., Merino J., Sun Q., Fito M., Salas-Salvado J. (2017). Dietary polyphenols, Mediterranean diet, prediabetes, and type 2 diabetes: a narrative review of the evidence. *Oxidative Medicine and Cellular Longevity*.

[13] Grosso G., Stepaniak U., Topor-Madry R., Szafraniec K., Pajak A. (2014). Estimated dietary intake and major food sources of polyphenols in the Polish arm of the HAPIEE study. *Nutrition*.

[14] Manach C., Williamson G., Morand C., Scalbert A., Remesy C. (2005). Bioavailability and bioefficacy of polyphenols in humans. I. Review of 97 bioavailability studies. *The American Journal of Clinical Nutrition*.

[15] Cory H., Passarelli S., Szeto J., Tamez M., Mattei J. (2018). The role of polyphenols in human health and food systems: a mini-review. *Frontiers in Nutrition*.

[16] Wang S., Moustaid-Moussa N., Chen L. (2014). Novel insights of dietary polyphenols and obesity. *The Journal of Nutritional Biochemistry*.

[17] Bose M., Lambert J. D., Ju J., Reuhl K. R., Shapses S. A., Yang C. S. (2008). The major green tea polyphenol, (-)-epigallocatechin-3-gallate, inhibits obesity, metabolic syndrome, and fatty liver disease in high-fat-fed mice. *The Journal of Nutrition*.

[18] Chiva-Blanch G., Badimon L. (2017). Effects of polyphenol intake on metabolic syndrome: current evidences from human trials. *Oxidative Medicine and Cellular Longevity*.

[19] Angulo P. (2002). Nonalcoholic fatty liver disease. *The New England Journal of Medicine*.

[20] Kotronen A., Yki-Jarvinen H. (2008). Fatty liver. *Arteriosclerosis, Thrombosis, and Vascular Biology*.

[21] Cui W., Chen S. L., Hu K. Q. (2010). Quantification and mechanisms of oleic acid-induced steatosis in HepG2 cells. *American Journal of Translational Research*.

[22] Buzzetti E., Pinzani M., Tsochatzis E. A. (2016). The multiple-hit pathogenesis of non-alcoholic fatty liver disease (NAFLD). *Metabolism*.

[23] (2016). EASL-EASD-EASO clinical practice guidelines for the management of non- alcoholic fatty liver disease. *Journal of Hepatology*.

[24] Augimeri G., Bonofiglio D. (2021). The Mediterranean diet as a source of natural compounds: does it represent a protective choice against cancer?. *Pharmaceuticals (Basel)*.

[25] Augimeri G., Montalto F. I., Giordano C. (2021). Nutraceuticals in the Mediterranean diet: potential avenues for breast cancer treatment. *Nutrients*.

[26] Korre M., Tsoukas M. A., Frantzeskou E., Yang J., Kales S. N. (2014). Mediterranean diet and workplace health promotion. *Current Cardiovascular Risk Reports*.

[27] Sofi F., Cesari F., Abbate R., Gensini G. F., Casini A. (2008). Adherence to Mediterranean diet and health status: meta-analysis. *BMJ*.

[28] Anania C., Perla F. M., Olivero F., Pacifico L., Chiesa C. (2018). Mediterranean diet and nonalcoholic fatty liver disease. *World Journal of Gastroenterology*.

[29] Rodriguez-Ramiro I., Vauzour D., Minihane A. M. (2016). Polyphenols and non-alcoholic fatty liver disease: impact and mechanisms. *The Proceedings of the Nutrition Society*.

[30] Boccellino M., D'Angelo S. (2020). Anti-obesity effects of polyphenol intake: current status and future possibilities. *International Journal of Molecular Sciences*.

[31] D'Angelo S. (2020). Current evidence on the effect of dietary polyphenols intake on brain health. *Current Nutrition & Food Science*.

[32] Williamson G., Holst B. (2008). Dietary reference intake (DRI) value for dietary polyphenols: are we heading in the right direction?. *The British Journal of Nutrition*.

[33] Perez-Jimenez J., Fezeu L., Touvier M. (2011). Dietary intake of 337 polyphenols in French adults. *The American Journal of Clinical Nutrition*.

[34] Tresserra-Rimbau A., Medina-Remón A., Pérez-Jiménez J. (2013). Dietary intake and major food sources of polyphenols in a Spanish population at high cardiovascular risk: the PREDIMED study. *Nutrition, Metabolism, and Cardiovascular Diseases*.

[35] Augimeri G., Galluccio A., Caparello G. (2021). Potential antioxidant and anti-inflammatory properties of serum from healthy adolescents with optimal Mediterranean diet adherence: findings from DIMENU cross-sectional study. *Antioxidants (Basel)*.

[36] Galluccio A., Caparello G., Avolio E. (2021). Self-perceived physical activity and adherence to the Mediterranean diet in healthy adolescents during COVID-19: findings from the DIMENU pilot study. *Healthcare*.

[37] Morelli C., Avolio E., Galluccio A. (2021). Nutrition education program and physical activity improve the adherence to the Mediterranean diet: impact on inflammatory biomarker levels in healthy adolescents from the DIMENU longitudinal study. *Frontiers in Nutrition*.

[38] Morelli C., Avolio E., Galluccio A. (2020). Impact of vigorous-intensity physical activity on body composition parameters, lipid profile markers, and irisin levels in adolescents: a cross-sectional study. *Nutrients*.

[39] WHO (1995). Physical status : the use of and interpretation of anthropometry , report of a WHO expert committee.

[40] WHO (2010). Global Recommendations on Physical Activity for Health.

[41] Garcia Cabrera S., Herrera Fernandez N., Rodriguez Hernandez C., Nissensohn M., Roman-Vinas B., Serra-Majem L. (2015). Kidmed test; prevalence of low adherence to the Mediterranean diet in children and young; a systematic review. *Nutrición Hospitalaria*.

[42] Kapolou A., Karantonis H. C., Rigopoulos N., Koutelidakis A. E. (2021). Association of mean daily polyphenols intake with Mediterranean diet adherence and anthropometric indices in healthy Greek adults: a retrospective study. *Applied Sciences*.

[43] Neveu V., Perez-Jimenez J., Vos F. (2010). Phenol-explorer: an online comprehensive database on polyphenol contents in foods. *Database*.

[44] Rothwell J. A., Perez-Jimenez J., Neveu V. (2013). Phenol-Explorer 3.0: a major update of the Phenol-Explorer database to incorporate data on the effects of food processing on polyphenol content. *Database*.

[45] Castro-Barquero S., Tresserra-Rimbau A., Vitelli-Storelli F. (2020). Dietary polyphenol intake is associated with HDL-cholesterol and a better profile of other components of the metabolic syndrome: a PREDIMED-plus sub-study. *Nutrients*.

[46] Stratil P., Klejdus B., Kuban V. (2006). Determination of total content of phenolic compounds and their antioxidant activity in vegetables--evaluation of spectrophotometric methods. *Journal of Agricultural and Food Chemistry*.

[47] Benzie I. F., Strain J. J. (1996). The ferric reducing ability of plasma (FRAP) as a measure of "antioxidant power": the FRAP assay. *Analytical Biochemistry*.

[48] Blois M. S. (1958). Antioxidant determinations by the use of a stable free radical. *Nature*.

[49] Rovito D., Gionfriddo G., Barone I. (2016). Ligand-activated PPAR*γ* downregulates CXCR4 gene expression through a novel identified PPAR response element and inhibits breast cancer progression. *Oncotarget*.

[50] Godos J., Rapisarda G., Marventano S., Galvano F., Mistretta A., Grosso G. (2017). Association between polyphenol intake and adherence to the Mediterranean diet in Sicily, southern Italy. *NFS Journal*.

[51] Hooper L., Kroon P. A., Rimm E. B. (2008). Flavonoids, flavonoid-rich foods, and cardiovascular risk: a meta-analysis of randomized controlled trials. *The American Journal of Clinical Nutrition*.

[52] Liu Y. J., Zhan J., Liu X. L., Wang Y., Ji J., He Q. Q. (2014). Dietary flavonoids intake and risk of type 2 diabetes: a meta-analysis of prospective cohort studies. *Clinical Nutrition*.

[53] Wisnuwardani R. W., De Henauw S., Androutsos O. (2019). Estimated dietary intake of polyphenols in European adolescents: the HELENA study. *European Journal of Nutrition*.

[54] Davis C., Bryan J., Hodgson J., Murphy K. (2015). Definition of the Mediterranean diet; a literature review. *Nutrients*.

[55] Feldman F., Koudoufio M., Desjardins Y., Spahis S., Delvin E., Levy E. (2021). Efficacy of polyphenols in the Management of dyslipidemia: a focus on clinical studies. *Nutrients*.

[56] Bo S., Ciccone G., Castiglione A. (2013). Anti-inflammatory and antioxidant effects of resveratrol in healthy smokers a randomized, double-blind, placebo-controlled, cross-over trial. *Current Medicinal Chemistry*.

[57] Castilla P., Echarri R., Davalos A. (2006). Concentrated red grape juice exerts antioxidant, hypolipidemic, and antiinflammatory effects in both hemodialysis patients and healthy subjects. *The American Journal of Clinical Nutrition*.

[58] Martinez-Lopez S., Sarria B., Sierra-Cinos J. L., Goya L., Mateos R., Bravo L. (2014). Realistic intake of a flavanol-rich soluble cocoa product increases HDL-cholesterol without inducing anthropometric changes in healthy and moderately hypercholesterolemic subjects. *Food & Function*.

[59] Tsang C., Smail N. F., Almoosawi S., McDougall G. J. M., Al-Dujaili E. A. S. (2018). Antioxidant rich potato improves arterial stiffness in healthy adults. *Plant Foods for Human Nutrition*.

